# Sustained Improvement in Quality of Patient Handoffs After Orthopaedic Surgery I-PASS Intervention

**DOI:** 10.5435/JAAOSGlobal-D-22-00079

**Published:** 2022-09-06

**Authors:** Derek S. Stenquist, Caleb M. Yeung, Hannah J. Szapary, Laura Rossi, Antonia F. Chen, Mitchel B. Harris

**Affiliations:** From the Harvard Combined Orthopaedic Residency Program, Boston, MA (Dr. Stenquist, Dr. Yeung); the Harvard Medical School, Boston, MA (Szapary); the Department of Orthopaedic Surgery, Massachusetts General Hospital, Boston, MA (Dr. Rossi, Dr. Harris); and the Department of Orthopaedic Surgery, Brigham and Women's Hospital, Boston, MA (Dr. Chen).

## Abstract

The I-PASS tool has been shown to decrease medical errors in patient handoffs in nonorthopaedic surgery fields. We prospectively studied the implementation of a version of this handoff tool modified for orthopaedic surgery patients in an academic practice at two level I trauma centers.

**Methods:**

This was a prospective study of a multicenter handoff improvement program. Handoffs were evaluated preintervention and at 1, 6, 9, and 18 months postintervention for key data elements defined by I-PASS. Rates of adverse clinical outcomes were compared before and after the handoff intervention.

**Results:**

Seven hundred five electronic patient handoffs were analyzed. From preintervention to the 18-month time point, notable improvement was observed in 8 of 9 targeted quality elements. In Poisson regression analysis, adherence to the standardized handoff format was sustained at markedly improved levels throughout all postintervention time points. No statistically significant differences were observed between rates of 30-day readmission, 90-day readmission, urinary tract infection, pulmonary embolism/deep vein thrombosis, surgical site infection, or delirium before and after the intervention.

**Conclusion:**

Introduction of an orthopaedic-specific I-PASS tool produced sustained adherence from a group of over 50 orthopaedic providers. Objective quality of handoffs improved markedly as defined by the I-PASS standard, and 86% of the providers supported the ongoing use of the tool. Despite the improvement in handoff quality, we were unable to demonstrate a notable change in measured clinical outcomes. Methods for the development and implementation of the orthopaedic-specific I-PASS tool are described. Orthopaedic residency programs should consider using a version of I-PASS to standardize care.

Medical errors are a leading cause of death among Americans.^1^ Transitions of patient care from one provider or team to another, also known as “handoffs,” are a risk factor for medical error.^2,3^ Interventions to improve and standardize communication during handoffs have been shown to reduce medical errors.^1,2^ The high volume of surgical patient care at many tertiary centers requires efficient and effective handoffs to minimize medical errors and optimize patient care.^3^

As elective procedures continue to shift to high-volume hospitals,^4^ orthopaedic surgery residents and advanced practice providers (APPs) are often tasked with providing perioperative care for complex trauma patients and elective surgery patients as part of one integrated orthopaedic surgery service line. In addition, many orthopaedic services have established geriatric comanagement services, resulting in more medically complex geriatric patients admitted to the orthopaedic service.^5^ Despite the increase in the number and complexity of patients on orthopaedic surgical services,^6^ very few studies have examined the best practices for handoffs in orthopaedic surgery. Moreover, duty-hour restrictions are estimated to have increased the number of patient handoffs by 130 to 200%,^2^ and the handoff phase of care is known to be a time of high error and lost information.^2,3,6,7^

Previous studies on orthopaedic surgery handoffs have described the development of handoff criteria de novo through surveys and focus groups.^8,9^ Rather than designing a new tool, we chose to adapt the I-PASS handoff template, which has been shown to decrease medical errors and preventable adverse events in nonorthopaedic surgery fields.^1^ The I-PASS tool includes the following quality elements: Illness severity, Patient summary, Action list, Situational awareness, Synthesis by receiver.^1,6^ The adoption of I-PASS was previously shown to be less consistent in surgical fields than in medicine and pediatrics,^6^ and its adaptation for orthopaedic surgery has not been previously described.

We administered a needs assessment to identify deficiencies in the existing handoff system at two level 1 trauma centers and then performed a prospective I-PASS intervention for orthopaedic surgery. Objective handoff quality and clinical outcomes were evaluated and compared before and after the intervention. The purpose of this study was to adapt the I-PASS tool for orthopaedic surgery and then determine the effectiveness and sustainability of a handoff improvement intervention by assessing the objective quality of patient handoffs over time.

## Methods

### Study Design

We conducted a prospective multicenter intervention study on inpatient orthopaedic surgery units at two level 1 trauma centers within a single large orthopaedic residency training program. Appropriate IRB approval was obtained for the conduction of this study.

#### Needs Assessment

In April 2019, a needs assessment survey about patient handoff communication was sent to all orthopaedic surgery residents and APPs (N = 74). Providers were asked to subjectively evaluate both the quality of patient handoffs and the effect of handoff quality on patient care. An abbreviated version of the survey is provided in Appendix 1, http://links.lww.com/JG9/A233. To objectively evaluate preintervention handoff quality, confidential orthopaedic surgery department safety reports for January 2017 to April 2018 were qualitatively analyzed. All reports were read and categorized according to previously established safety report criteria.

#### I-PASS Tool Development

**Figure 1:**
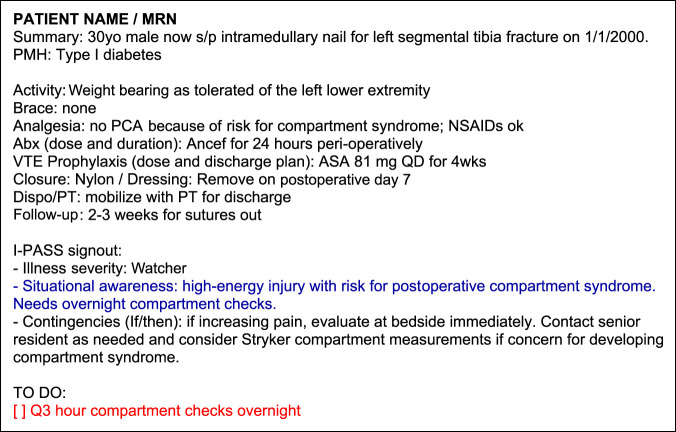
Example of an abbreviated OrthoPass signout using the example of a young male patient with a high-energy tibial shaft fracture at risk of developing postoperative compartment syndrome.

After completion of the needs assessment, an interdisciplinary team was assembled to adapt the I-PASS tool for orthopaedic surgery. The team consisted of two orthopaedic surgery residents, the Chairman of orthopaedic surgery and an RN/PhD who served as the Quality and Patient Safety Program Manager for the department of orthopaedic surgery. The I-PASS for orthopaedic surgery tool was developed through an iterative process based on best evidence from the literature.^6,10-12^ It incorporated all key I-PASS elements but streamlined the I-PASS handoff for use in surgery (Figure 1). The adapted handoff tool called “OrthoPass” was built into the electronic medical record (EMR) as a “SmartPhrase” template to allow all orthopaedic providers to access and use the same content.

#### Focus Groups

Two focus groups were held with residents and APPs before the intervention to demonstrate the use of OrthoPass and solicit feedback. Changes were made in an iterative fashion based on provider feedback. For example, anticoagulation plan and antibiotic plan are not specifically part of I-PASS but were added as quality metrics because of the feedback received during focus groups. Residents and APPs reported poor communication about dosing and duration of postoperative antibiotics and details of the postoperative anticoagulation plan, so these were added to the tool to tailor I-PASS for orthopaedic surgery.

#### Intervention

In May 2019, because of the ongoing COVID-19 pandemic, the intervention was initiated through a secure electronic correspondence notifying all orthopaedic surgery residents, fellows, and APPs to begin using the OrthoPass handoff tool. Detailed instructions were provided. Providers were asked specifically to use the template for all postoperative patients or surgical consult patients belonging to the following services: orthopaedic trauma, hand, arthroplasty, spine, and foot and ankle. All providers were notified that adherence would be assessed by way of written handoff monitoring throughout the study period. A unique I-PASS handoff document was created by a resident or APP for each postoperative or surgical consult patient immediately after surgery or on consultation using the EMR “SmartPhrase” template. The resident or APP caring for each patient updated the electronic handoff document before each transition of care (typically daily or twice daily within a night float system). Therefore, the written handoff served as a structured, live document updated at each transition of care and reviewed online during handoff.

#### OrthoPass Maintenance

Maintenance of adherence was achieved in several ways. The Chairman of orthopaedic surgery notified all residents, fellows, and APPs of the intervention to demonstrate support for OrthoPass. Periodic e-mail notifications were sent to providers reminding them to use the OrthoPass template at one month, 3 months, and 6 months after the intervention. Feedback solicited throughout the study period was promptly acknowledged and acted upon to encourage provider buy-in. For example, suggested changes to the tool could be implemented in the EMR template and were then immediately effective for all providers using the OrthoPass “SmartPhrase.” Individual providers who were noncompliant with the template use were emailed politely on a case-by-case basis to encourage the use of the OrthoPass template. Finally, throughout the two-year study period, the OrthoPass-led residents attended all new resident and fellow training sessions virtually or in person to introduce the OrthoPass template and emphasize the importance of patient handoffs.

### Study Outcomes

#### Written Handoff Quality

Objective handoff quality was evaluated and compared preintervention and at 1 (pilot phase), 6, 9, and 18 months postintervention. Handoffs were evaluated for the presence of the following key data elements defined by I-PASS: two patient identifiers, illness severity, medical history, action list, situational awareness, and contingencies. The quality of communication regarding postoperative antibiotic and anticoagulation plans was also evaluated.

#### Postintervention Provider Survey

A postintervention survey was administered to providers at 6 months after the start of the OrthoPass initiative. Providers were asked to subjectively evaluate the effect of the OrthoPass program on handoff quality and patient safety. A copy of the postintervention survey is provided in Appendix 2, http://links.lww.com/JG9/A234.

### Statistical Analysis

A chi square test or Fisher exact test was used for categorical analysis with a *P*-value of <0.05 as the criteria for statistical significance. To trend rates of adherence over time, Poisson regression analysis was conducted using sequential Sidak adjustment for multiple comparisons, with the preintervention group means used as the referent for each respective regression. Statistical analyses were conducted in SPSS v27.0 (IBM Corporation, Armonk, NY).

### Clinical Outcomes

We compared rates of adverse clinical outcomes before and after the handoff intervention including 30-day readmission, 90-day readmission, urinary tract infection (UTI), pulmonary embolism/deep vein thrombosis (DVT), surgical site infection, and delirium.

## Results

### Needs Assessment

Fifty-six orthopaedic providers completed the preintervention needs assessment survey (76% response rate). Most of the providers reported that the quality of existing handoffs was inadequate. 59% were “sometimes” or “often” uncertain about making a clinical decision because they lacked patient information from a handoff. Seventy-one percent reported that they had “sometimes” or “often” received an inadequate handoff about a recently admitted patient. 73% reported that important patient care information was “sometimes” or “often” lost during shift changes. Respondents reported that handoffs for patients admitted to a service other than trauma were often or always adequate only 25% of the time.

More than 50% of the respondents reported that problems with handoffs had resulted in increased length of stay, delay in diagnosis or treatment, need for additional testing or monitoring, and patient discomfort or pain. Another 40% reported that problems with handoffs had caused medication errors. Providers were also asked to describe the most serious harmful event they witnessed related to handoff quality. 77% attributed the harmful event to the omission or lack of adequate essential information in a handoff. 91% of the respondents stated that they would support a standardized electronic handoff template to organize and catalog essential information to be used during verbal handoffs.

Seventy-four patient safety reports were analyzed from the 15 months preceding the intervention. 40% of the orthopaedic safety reports involved a breakdown in communication between inpatient providers. The most common communication issues included confusion about the responsible provider during transitions of care (22%) and inadequate handoff information during transitions of care (17%). According to the patients filing the safety reports, these communication failures resulted in delayed responses, increased risk, and failure to rescue patients from harm.

### Written Handoff Quality

**Figure 2:**
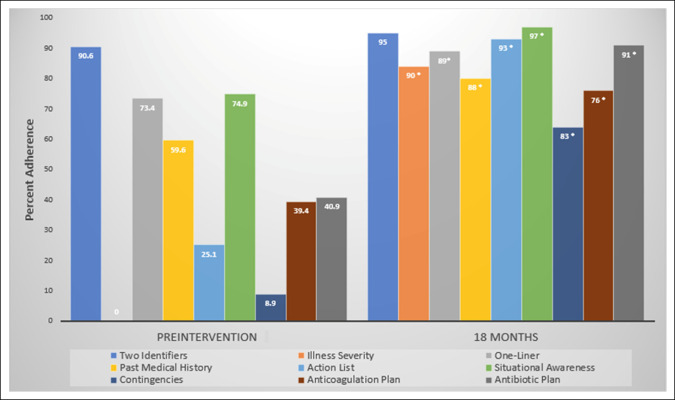
Graph showing key I-PASS elements included in patient handoffs before the intervention compared with 18 months after the intervention. *P = 0.002 for one-liner, *P* < 0.001 for all others.

**Figure 3:**
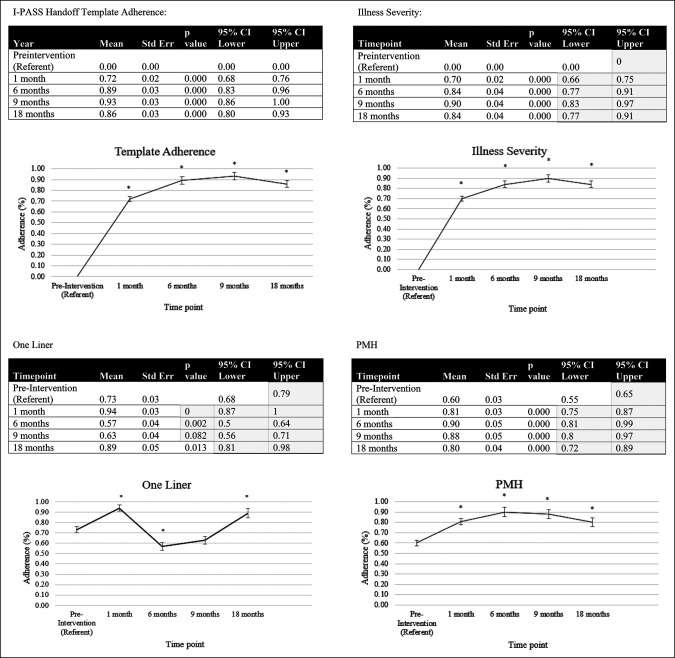

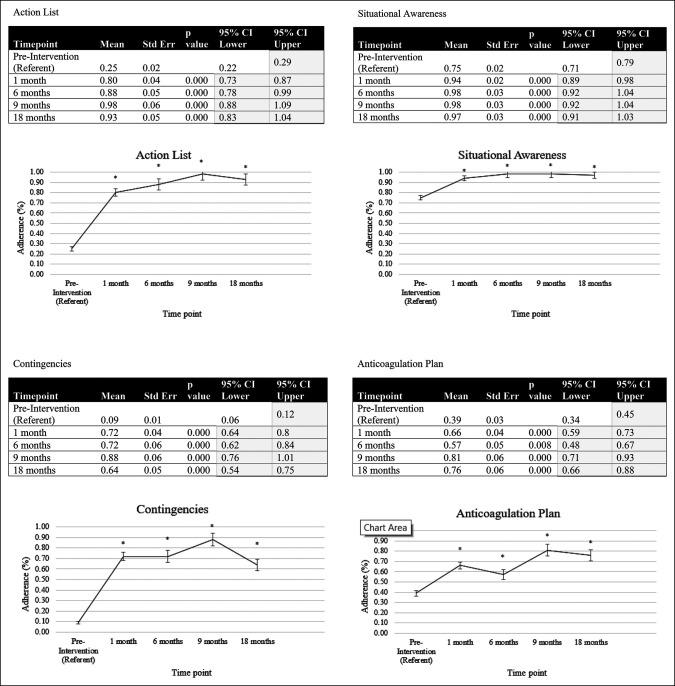

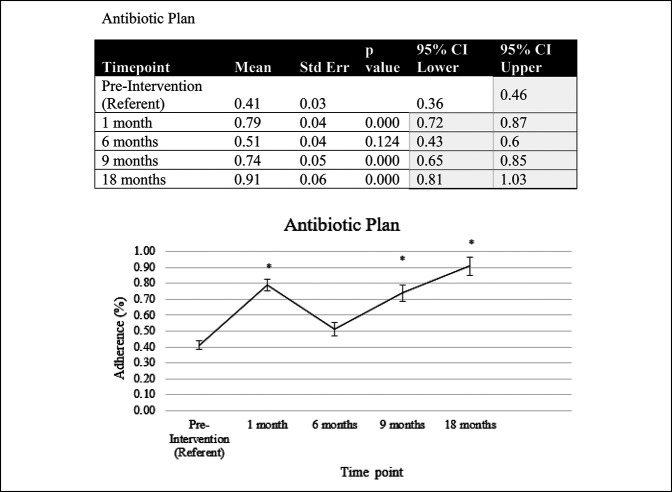
Chart showing Poisson regression analyses with the preintervention group means used as the referent for each respective regression. Improvements in handoff quality were sustained over time at statistically significant levels.

A total of 705 electronic patient handoffs were analyzed between May 2019 and December 2020. A pre hoc power calculation for the pilot study of 1-month outcomes demonstrated that 304 total handoff observations (152 per group) were needed to have 80% power to detect a change of 5% with an alpha error of 0.05. Two hundred three consecutive preintervention handoffs were compared with 202 postintervention handoffs in the pilot phase. One hundred handoffs were then analyzed at each of the three subsequent time points for a total of 705 handoffs analyzed. From preintervention to the 18-month time point, significant improvement was noted in 8 of 9 targeted quality elements (illness severity, medical history, action list, situational awareness, contingencies, anticoagulation plan, and antibiotic plan, *P* = 0.002 for one liner, *P* < 0.001 for all others) (Figure 2). When trended over time, Poisson regression analyses demonstrated that these improvements were sustained over time at statistically significant levels (Figure 3).

### Postintervention Survey

Fifty-four providers completed the postintervention survey (73% response rate). Seventy percent reported subjective improvement in handoff communication and patient safety after the intervention; 60% stated that the intervention reduced patient errors and near misses in their opinion. Ninety-six percent of the providers supported the implementation of the OrthoPass tool as a requirement for all new trainees.

### Clinical Outcomes

A patient data repository system was used to retrieve records for 1012 consecutive patients admitted preintervention and 972 consecutive patients admitted 6 months postintervention. No statistically significant differences were observed between rates of 30-day readmission, 90-day readmission, UTI, pulmonary embolism/DVT, surgical site infection, or delirium before and after the intervention.

## Discussion

In 2000, the landmark Institute of Medicine report “To Err is Human” estimated that up to 98,000 deaths occur annually from preventable medical errors. In a subsequent analysis, Makary and colleagues estimated up to 250,000 annual deaths from medical errors using total US hospital admissions in 2013 and extrapolating from studies published since the Institute of Medicine report. The handoff phase of care is a particularly high-risk period for medical errors,^2,13^ especially surrounding surgical events.^7^ A large body of research in the medical literature has been devoted to improving communication during transitions of care.

The New England Journal of Medicine article on I-PASS published in 2014 established a new standard for handoff communication. Researchers demonstrated that implementation of a comprehensive handoff improvement program using the I-PASS template reduced medical errors by 23% and adverse events by 30% across nine hospitals studied. More recently, Shahian and colleagues^6^ described their experience implementing I-PASS at a large academic medical center. They noted progressive but nonuniform adoption of I-PASS across services within the hospital. In particular, uptake of I-PASS on surgical services was less consistent than in pediatrics or internal medicine. Surgical residents reported that they covered a large number of postoperative patients who were often completely stable, and they had insufficient time to do a formal I-PASS handoff on each patient. The authors encouraged surgical services to appropriately adapt the basic I-PASS structure to their needs, especially for straightforward patients.^6^

The literature on handoff communication, specifically for orthopaedic surgery patients, is limited. However, handoffs are an important component of all residency training. The Joint Commission has included standardization of handoff communications among its National Patient Safety Goals since at least 2006, and accrediting bodies for graduate medical education require training in handoff communication. Handoff communication may be particularly important surrounding surgical events because they represent complex transitions of care from an initial caregiver (surgeon or senior resident) to a new team (inpatient APP or junior resident).^7,14^ In addition, duty-hour restrictions have increased the number of handoffs, leading to decreased continuity of care and more opportunities for error.^2^

Prior efforts to improve patient handoffs in orthopaedics have described the development of handoff criteria de novo through surveys and focus groups. Sleiman et al^15^ conducted a review of current literature on surgical checklists and handoff tools for orthopaedic surgery. They emphasized the potential for checklists to improve cost-effectiveness and reduce both mortality and complications, but they noted a lack of orthopaedic-specific tools. LeBlanc et al developed a preoperative handoff checklist for orthopaedic trauma patients based on expert opinion. Notably, 94% of orthopaedic providers surveyed in the study agreed that handoffs should be taught. Gagnier et al^9^ met with stakeholders to develop an orthopaedic-specific handoff template. They analyzed rates of adverse events through a chart review of 120 patients before and after implementation and found a nonsignificant reduction in events per person. Their template included many of the same orthopaedic-specific components as OrthoPass, such as antibiotic coverage, DVT prophylaxis, and weight-bearing status, but did not incorporate I-PASS elements, such as illness severity or contingency planning.^1^

We were able to achieve sustained adherence with an adapted version of I-PASS for orthopaedic surgery in part by anticipating barriers to adoption. We predicted that the most difficult aspects of improving I-PASS adherence on our busy surgical service would relate to the same hurdles described by Shahian et al. We sought to encourage culture change while not disrupting the existing workflows of busy residents. We deliberately incorporated many of the best practices described by Shahian et al during their experience implementing I-PASS: administrative and clinical leadership support, EHR templates for I-PASS, handover observations, resident I-PASS champions, and solicitation of caregiver feedback and suggestions. For example, the electronic OrthoPass template worked well for stable postoperative patients who did not need an extended verbal handoff, and this fit seamlessly into the established workflows.

We also anticipated worse adherence among fellows compared with residents because fellows are “visitors” for one year and may not adapt quickly to institutional culture. Satisfactory adherence to the template among both residents and fellows in this study is likely the result of a targeted strategy of education during both resident and fellow orientation and frequent reminders after handoff monitoring. Anecdotally, we found that prompt responses to resident and staff feedback and implementation of appropriate suggested changes served to increase buy-in. Another key was resident champion leadership. Rather than attempting a top-down administrative implementation, the intervention relied on a resident-designed template because residents are uniquely poised to understand the demands of caring for perioperative patients during surgical training.

Although OrthoPass markedly improved adoption of I-PASS and handoff quality, certain components were notably more difficult to implement. Only 1.6% of the patients were categorized as “watchers,” which likely underestimates the prevalence of patients in this category. However, a formal definition of “stable,” “watcher,” and “unstable” has never been established in the literature for any field. Future studies should attempt to objectively define these categories for orthopaedic surgery patients because we relied on expert opinion to create orthopaedic-specific criteria. We also noted a decline in adherence over time with the use of the “contingency” category, which is intended to provide “if/then” statements to help receiving clinicians identify and address anticipated problems. This may be because of a lack of familiarity with the term contingency planning or because of the stable nature of most postoperative orthopaedic surgery patients.

Despite the improvement in the handoff structure and content according to the I-PASS quality elements, we did not observe any notable change in the clinical outcomes measured before and after the intervention. The clinical outcomes obtained from a patient data repository were intended to serve as surrogates for preventable adverse events. It is possible that the handoff intervention program was not comprehensive enough to result in a measurable improvement in these clinical outcomes. The analysis may also be underpowered; our study examined outcomes for 1984 patients before and after intervention compared with more than 10,000 in the NEJM I-PASS study.^1^ Outcomes such as readmission rates are also subject to confounding by many factors besides in-hospital handoff communication. Finally, some adverse events or near misses are not captured by the clinical outcomes we measured, and the benefits of progressive handoff culture change may take longer than 6 months to accrue.

Our study has several important limitations. For our primary outcome, we relied on previously established objective measures of handoff quality using the same methods as the NEJM article on I-PASS.^1^ Although this method evaluates the quality and organization of handoffs, it is unable to measure the accuracy of the handoffs. Moreover, we were not able to duplicate the NEJM article measurements of adverse events and medical errors because of the large associated costs and logistic challenges. For example, the NEJM study on I-PASS relied on trained research nurses to review all medical records and orders on the study unit 7 days per week and then report observed and suspected errors. We also used provider surveys preintervention and postintervention, which are subject to recall bias.

## Conclusions

This prospective study analyzed the effect of an I-PASS handoff improvement program on the quality of handoff communication for orthopaedic inpatients. The introduction of an orthopaedic-specific I-PASS tool produced a high, sustained adherence rate from a group of over 50 orthopaedic providers. Objective quality of handoffs improved markedly as defined by the I-PASS standard. Nearly 90% of the providers supported the ongoing use of the tool, with 70% stating that communication and patient safety improved. Despite the improvement in handoff quality, we were unable to demonstrate a notable change in measured clinical outcomes. Methods for the development and implementation of the orthopaedic-specific I-PASS tool are described. Orthopaedic residency programs should consider using a version of I-PASS to standardize care.
